# Biocontrol Effect of *Bacillus velezensis* D7-8 on Potato Common Scab and Its Complete Genome Sequence Analysis

**DOI:** 10.3390/microorganisms13040770

**Published:** 2025-03-28

**Authors:** Yu Jiang, Pengfei He, Huihui Kong, Pengbo He, Yixin Wu, Guowen Tang, Ping Tang, Yining Di, Xingyu Li, Lufeng Liu, Shahzad Munir, Yueqiu He

**Affiliations:** State Key Laboratory for Conservation and Utilization of Bio-Resources in Yunnan, Yunnan Agricultural University, Kunming 650201, China

**Keywords:** potato common scab, *Bacillus*, comparative genomics, structural variation, gene clusters, machining learning

## Abstract

Potato common scab, caused by *Streptomyces* species, is a widespread soil-borne disease that poses a significant threat to potato cultivation globally. In this study, a *Bacillus velezensis* D7-8 strain was isolated from a potato. This endophytic bacterium exhibited broad-spectrum antifungal activity, and pot trials demonstrated that the D7-8 strain effectively controlled potato common scab with an efficacy of 42.07%. The complete genome sequence of the D7-8 strain was sequenced and subsequently identified as *B. velezensis* through multiple bioinformatic methods, primarily through structural variation analysis of whole-genome sequences. The machine learning method predicted that the expression profiles of colinear genes among closely related *Bacillus* species were highly consistent. Metabolite analysis of crude extracts using ultra-high-performance liquid chromatography coupled with quadrupole-Orbitrap high-resolution mass spectrometry (UPLC-Q-Exactive HRMS) revealed that D7-8 produces bioactive compounds, including surfactin and fengycin, both known for their antimicrobial properties. This study elucidates the antagonistic effect of *B. velezensis* D7-8 against *Streptomyces acidiscabies* and provides a valuable reference for future research on accurate microbial identification.

## 1. Introduction

Potato common scab is a significant disease affecting potato crops worldwide [1]. This disease results in the formation of corky lesions on potato tubers, significantly reducing their market value and leading to considerable economic losses. The primary impact of potato common scab is the development of unsightly lesions on tubers, which can range from superficial blemishes to deep pitting. These defects render the affected potatoes unsuitable for fresh consumption and processing, further exacerbating financial losses [2]. In addition, this disease can compromise tuber storability, as the lesions serve as entry points for secondary infections by other pathogens, increasing post-harvest losses [1,2].

Previous research has indicated that potato common scab is caused by a group of Gram-positive, filamentous bacteria belonging to the genus *Streptomyces* [3]. The most commonly implicated species include *Streptomyces scabies*, *S. acidiscabies*, and *S. turgidiscabies* [4]. *S. scabies* is the most prevalent and well-characterized pathogen [3,5]. It produces a variety of phytotoxins, including thaxtomin, which disrupts plant cell wall synthesis and contributes to scab lesion formation [6,7]. *S. acidiscabies* is associated with acid scab, a common scab variant found in acidic soils. While it shares pathogenic mechanisms with *S. scabies*, it thrives in low-pH environments [8]. Meanwhile, *S. turgidiscabies* is another significant pathogen linked to potato common scab. It produces thaxtomin-like toxins and is responsible for raised, corky, crusty lesions on potato tubers [9]. These *Streptomyces* bacteria become active and infect developing potato tubers through natural openings or wounds. Once established, they colonize the tuber surface and secrete thaxtomin toxins that interfere with plant cell wall synthesis and trigger the formation of characteristic scab lesions [2,5]. Environmental factors play a pivotal role in the epidemiology of common scab. Variables such as soil moisture, temperature, pH, and organic matter content significantly influence disease severity and incidence. High soil moisture during the early stages of tuber development suppresses bacterial infection and reduces disease occurrence, whereas dry conditions promote bacterial activity and scab formation [10].

Chemical agents or pesticides have traditionally been applied to manage potato common scab, including dazomet, chloropicrin, hydroquinone, mercuric chloride, methanal, muriatic acid, metam-sodium, mancozeb, phenylpropithiazole, fluridine, and pesticide mixtures [2,11,12,13,14,15]. However, the results showed that chemical agents or pesticides can only partially suppress pathogen growth during potato development and are insufficient to completely prevent or eradicate this disease. Biocontrol methods have been studied in parallel with chemical control for managing potato common scab. Biocontrol agents, like *Bacillus* [16], *Trichoderma* [17], *Pseudomonas* [18], and *Gliocladium* [19], have been developed for managing potato common scab, and these microbes demonstrate better control efficacy than chemical agents or pesticides, especially for the *Bacillus* genus [16]. As common environmental and endophytic bacteria in potatoes, *Bacillus* species hold great potential for effectively suppressing potato common scab. Genetic improvement is a promising strategy to enhance the biocontrol efficacy of these endophytic bacteria [20]. Bacterial genomes, characterized by their small size, low repeat content, and lack of introns [21,22,23], offer a more accessible genetic editing platform than plants and animals. However, obtaining complete and highly accurate genome sequences is a crucial first step in unlocking their full potential. Next-generation sequencing technologies from the Illumina, PacBio, and Nanopore platforms provide highly efficient approaches for obtaining complete and accurate genome sequences, especially as sequencing costs continue to decrease [10,24].

In this study, we conducted complete genome sequencing and analysis of *Bacillus* D7-8, an endophytic bacterium isolated from the potato variety ZM1. This strain exhibited potent and broad-spectrum antagonistic activity against various pathogenic fungi and bacteria, particularly those of the *Streptomyces* genus responsible for potato common scab. Through comprehensive bioinformatic analysis, including Average Nucleotide Identity (ANI), phylogenetics, and comparative genomics, D7-8 was identified as *Bacillus velezensis*. Key genes associated with secondary metabolite biosynthesis were subsequently characterized, and the expression patterns of these genes were predicted using a machine learning approach. The resulting secondary metabolites were analyzed using HPLC-MS. These insights enhance our understanding of the antimicrobial mechanisms of this unique *B. velezensis* strain and may contribute to developing novel biocontrol strategies.

## 2. Materials and Methods

### 2.1. Pathogens Resources and Culture Medium for the Experiment

The pathogens: *Alternaria solani* (PV366807), *Phytophthora nicotianae* (PV335816), *Fusarium graminearum* (PP515275.1), *Sclerotium rolfsii* (OR507253.1), *Rhizoctonia solani* (LC858425.1), and *Epicoccum latusicollum* (OR121534.1) were maintained in Molecular Plant Pathology laboratory, while *Streptomyces acidiscabies* (PV366806) was provided by the Potato Disease Laboratory of Yunnan Agricultural University, Kunming, China.

LB (Luria–Bertani) medium is prepared by mixing 10 g of peptone, 5 g of yeast extract, and 10 g of NaCl in 1 L of distilled water. For solid media, 15 g of agar is added. The pH is adjusted to 7.0 using NaOH. To prepare PDA (potato dextrose agar) medium, 200 g of peeled potatoes are boiled in 1 L of distilled water for 30 min. The potato infusion is filtered through cheesecloth, and 20 g of glucose and 15 g of agar are added to the filtrate. The final volume is adjusted to 1 L with distilled water without pH adjustment. OA (oatmeal agar) medium is made by boiling 30 g of oatmeal in 1 L of distilled water for 30 min. The mixture is filtered through cheesecloth to remove solids, and 20 g of agar is added to the oatmeal filtrate. The final volume is restored to 1 L with distilled water without pH adjustment. All prepared media are autoclaved at 121 °C (15 psi) for 30 min before use.

### 2.2. Isolation of Endophytic Bacteria

The potato variety used in this study was ZM1, maintained at the Potato Disease Laboratory, Yunnan Agricultural University, China. Tuber surfaces were rinsed with tap water, air-dried, and sterilized by soaking in 75% ethanol for 5 min, followed by immersion in 2% (*w*/*v*) sodium hypochlorite (NaOCl) for 4 min [25]. The tubers were then rinsed six times with sterile distilled water. To confirm complete sterilization, 100 µL of the final rinse was plated on Luria–Bertani (LB) medium. All procedures were performed under a laminar flow hood. Surface-sterilized potato tubers were cut into 1 cm cubes and homogenized in a sterilized mortar and pestle with 9 mL of sterile water until a uniform paste was obtained. Serial dilutions (10^−1^ to 10^−5^) of the homogenate were prepared, and 100 µL of each dilution was plated on LB medium and incubated at 37 °C for 24 h. Individual colonies were examined for color and morphology, purified, and designated according to our previous study [26].

### 2.3. Antagonistic Screening of S. acidiscabies Strains

The 100 μL suspension of *S. acidiscabies* of 1 × 10^7^ CFU/mL was applied to the PDA medium. Endophytic bacteria were placed at the center of the plate using a sterile toothpick and incubated at 28 °C and 70% humidity for 3 days. The inhibition zone diameter for each bacterium was recorded, and 1 μL of LB broth was used as a control [27]. Each experiment was performed in triplicate.

### 2.4. Detection of Broad-Spectrum Fungistatic Ability of D7-8

Following a similar method, each pathogenic fungus was purified and cultured separately on potato dextrose agar (PDA) at 28 °C with 60% relative humidity for 7 days, except for *P. nicotianae*, which was incubated on oatmeal agar. An 8 mm fungal plug was excised using a hole punch and placed at the center of a PDA plate. Three equidistant points along intersecting center lines were inoculated with D7-8, while the remaining point served as a blank control. After 7 days of incubation, the inhibition zone of D7-8 against each pathogen was measured [28]. Each treatment was also performed in triplicate.

### 2.5. Pot Trials

The experiment was conducted in a greenhouse with a maximum temperature of 32 °C, a temperature difference of less than 10 °C between day and night, relative humidity of 50–70%, and a 12 h light period per day. Pre-sprouted potato seed tubers of uniform size and similar bud length were selected and planted in autoclaved, healthy soil, sourced from an environment free of potato common scab, with appropriate fertilization and watering. After 20 days of growth, each pot was inoculated with 100 mL of *S. acidiscabies* (10^8^ CFU/mL) via root irrigation. Seven days later, and again during the tuber formation stage, the plants were irrigated with 100 mL of the D7-8 microbial agent (10^8^ CFU/mL). After 120 days of growth, the plants were harvested and evaluated for disease indices. Each treatment included 8 plants, for a total of 16 potato plants. Treatment A was inoculated with *S. acidiscabies* only as a control check, while treatment B received both *S. acidiscabies* and D7-8 as a preventive treatment. Potato disease severity scale: Grade 1 (assigned value = 0): no visible spots on potato surface; Grade 2 (assigned value = 1): spots cover 0.1–5% of total potato area; Grade 3 (assigned value = 2): spots cover 5.1–12.5% of total potato area; Grade 4 (assigned value = 3): spots cover 12.6–25% of total potato area; Grade 5 (assigned value = 4): spots cover 25.1–50% of total potato area; and Grade 6 (assigned value = 5): spots cover >50% of total potato area. Disease index = ∑ (Number of potatoes per grade × Grade assigned value)/Total potatoes surveyed × Maximum grade assigned value × 100%. Treatment efficacy (%) was calculated as follows: Efficacy of treatment (%) = (Disease index of control group − Disease index of treatment group) × 100%/Disease index of control group [29].

### 2.6. Preliminary Molecular Identification of D7-8 Strain

Strain D7-8 was retrieved from glycerol stocks and purified by streaking onto LB agar, followed by incubation for 24 h. Single colonies were selected, and genomic DNA of D7-8 was extracted using a DNA extraction kit (Sangon Biotech Co., Ltd., Shanghai, China), following the manufacturer’s protocol. PCR amplification of the 16S rRNA and *rpoB* genes was performed using our previous method [30]. Phylogenetic analysis was conducted using MEGA 11.0, employing the maximum likelihood method with 1000 bootstrap replicates [31].

### 2.7. Genome Sequencing and Annotation of D7-8

According to the whole-genome sequencing protocol, purified D7-8 strains were cultured to the logarithmic phase, and bacterial cells were centrifuged at 16,000 rpm for 5 min. The harvested bacterial pellets were immediately treated with liquid nitrogen and then stored at −80 °C. Whole-genome sequencing was performed by Majorbio Co., Ltd. (Shanghai, China).

The whole genome of *Bacillus velezensis* D7-8 was de novo sequenced using the PacBio Sequel II Single-Molecule Real-Time (SMRT) platform with 20 kb insert libraries and the Illumina NovaSeq 6000 platform (Illumina, Inc.; San Diego, CA, USA) with 496 bp insert libraries. Low-quality reads were filtered using Fastp v0.20.0 [32] to obtain a high-quality clean dataset, which was subsequently assembled into a contiguous genome using Unicycler v0.4.8 and polished with the clean dataset from the Illumina platform, resulting in a complete genome with seamless chromosomes and plasmids [33]. Plasmid sequences were identified using PlasFlow v1.1 and annotated through BLAST v2.12.0 against the PLSDB dataset [34]. The completeness of the final assembly was assessed using BUSCO v5.7.1 with the bacteria_odb10 database [35].

A repeat sequence library was constructed to analyze the complement of tandem repeats and gain insights into their function and evolutionary significance in D7-8. Repeat sequences were predicted using RepeatMasker v3.3.0 and Tandem Repeat Finder (TRF), which were employed to identify homologous tandem and interspersed repeats [36,37].

Coding sequences (CDSs) were predicted for gene annotation using Glimmer, GeneMarkS, and Prodigal. The functions of protein-coding genes were annotated using multiple databases [38], including UniProt (Universal Protein Resource), NR (Non-Redundant Protein Database), Pfam (http://pfam.xfam.org/, accessed on 11 December 2024), GO (Gene Ontology), COG (Clusters of Orthologous Groups), and KEGG (Kyoto Encyclopedia of Genes and Genomes) (https://www.kegg.jp/, accessed on 10 December 2024). Additionally, tRNA, rRNA, and sRNA genes were identified using tRNAscan-SE [39], Barrnap (https://github.com/tseemann/barrnap, accessed on 10 December 2024), and Infernal [40], respectively. The completeness of gene prediction was assessed using BUSCO v5.7.1 with the bacteria_odb10 database [35].

For metabolite analysis, carbohydrate-active enzymes in the *Bacillus velezensis* D7-8 genome were identified using the CAZy database (http://www.cazy.org/, accessed on 17 December 2024). In contrast, gene clusters involved in secondary metabolite biosynthesis were predicted using antiSMASH (https://antismash.secondarymetabolites.org/, accessed on 11 December 2024).

### 2.8. Comparative Genomic Analysis of D7-8

Based on 31 housekeeping genes, the closest bacterial species were identified by comparison with a reference database, and a phylogenetic tree was constructed using the neighbor-joining method in MEGA 11.0 [30]. To further refine the species classification of D7-8, 45 reference genomes of *Bacillus* species, along with their annotation files, were retrieved from the National Center for Biotechnology Information (NCBI, https://www.ncbi.nlm.nih.gov/datasets/genome/?taxon=1386,55087, accessed on 8 December 2024). The reference genomes were highlighted in green for distinction in the NCBI genome database and were almost fully assembled at the complete or chromosome level.

Average Nucleotide Identity (ANI) analysis was performed using FastANI v1.34 [41] across 46 *Bacillus* genomes, including D7-8. Additionally, phylogenetic trees at the species level were constructed using OrthoFinder v3.0.1b1 [42], based on all protein sequences from each *Bacillus* species. Structural variations (SVs) were identified using MUMMER v3.1 [43] and SyRI v1.7.0 [44]. Furthermore, the potential expression pattern related to secondary metabolite biosynthesis gene clusters was assessed using TXpredict software [45].

### 2.9. Identification of Lipopeptide Compounds by UPLC-Q-Exactive HRMS

Preparation of crude lipopeptide extract: Following the method described above, the collection solution was adjusted to pH 2.0 using 10% hydrochloric acid, and the precipitate was collected by centrifugation at 12,000 rpm for 10 min at 4 °C. The precipitate was then incubated at 4 °C for 12 h. The appropriate quantity of methanol was added to facilitate the dissolution of the precipitate, which was then incubated at 4 °C for 12 h. The pH was adjusted to 7.0 with NaOH, after which the material was freeze-dried in a vacuum, redissolved in methanol, passed through a 0.22 μm filter membrane, and stored at 4 °C for later use [46].

The composition was analyzed using UPLC-Q-Exactive HRMS under the following conditions: The Thermo Scientific Accucore C18 column (2.1 mm × 100 mm, 2.6 μm packed) (Thermo Fisher Scientific; Waltham, MA, USA) was used with 0.1% formic acid–acetonitrile as mobile phase A and 0.1% formic acid–water as mobile phase B. The gradient elution mode was used, starting with 5% acetonitrile–0.1% formic acid–water and gradually increasing to 95%, with a duration of 45 min. The flow rate was set at 0.4 mL-min^−1^, and the injection volume was 3 μL. The column temperature was maintained at 35 °C throughout the analysis, and the mass-to-charge (*m*/*z*) detection range was set from 100 to 1500 [47].

### 2.10. Statistical Analysis

Statistical analysis of variance (ANOVA) was performed on the data using SPSS software (version 17.0, IBM, USA), and multiple comparisons were conducted to test the significance of differences between multiple samples using Duncan’s new complex polarity method. The difference was considered significant at *p* < 0.05.

## 3. Results

### 3.1. Inhibition and Control Effect of D7-8 on Potato Common Scab and Its Broad-Spectrum Fungal Inhibition Ability

**Figure 1:**
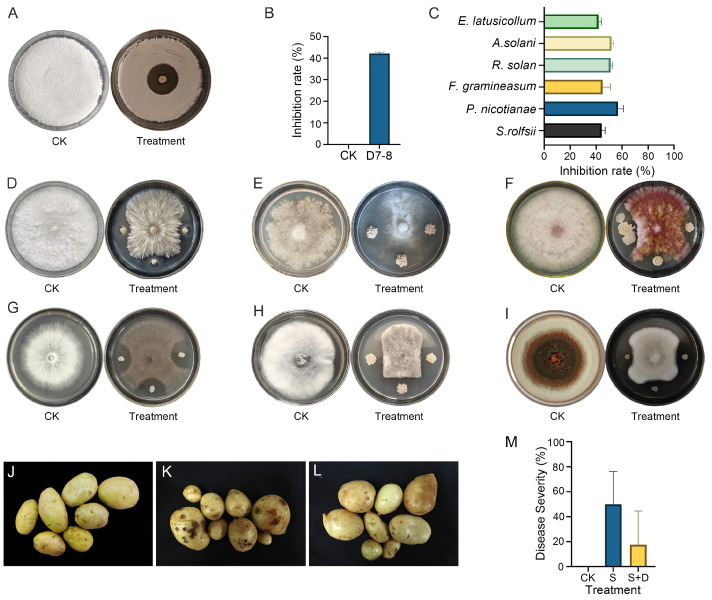
Broad-spectrum antimicrobial activities of D7-8 and its biocontrol effect against potato common scab: (**A**) inhibitory effect of D7-8 against *S. acidiscabies*; (**B**) inhibition ratios of D7-8 against *S. acidiscabies*; (**C**) statistic of inhibition ratios of D7-8 for each pathogen; (**D**) inhibitory effect of D7-8 against *S. rolfsii*; (**E**) inhibitory effect of D7-8 against *P. nicotianae*; (**F**) inhibitory effect of D7-8 against *F. graminearum*; (**G**) inhibitory effect of D7-8 against *R. solani*; (**H**) inhibitory effect of D7-8 against *A. solani*; (**I**) inhibitory effect of D7-8 against *E. latusicollum*; (**J**) CK in pot experiment; (CK); (**K**) inoculate *S. acidiscabies* only in pot experiment (S); (**L**) simultaneously inoculate *S. acidiscabies* and D7-8 in pot experiment (S + D); and (**M**) disease severity of D7-8 against potato scab in the pot experiment.

From potato tubers, we isolated a total of 41 strains, five of which had antagonistic activity (Table S1). Among them, D7-8 showed significant antagonistic activity against *S. acidiscabies*, with an inhibition rate of 42.07 ± 0.64% (Figure 1A,B). Therefore, we selected D7-8 for further testing.

Six crop pathogenic fungi (*A. solani*, *P. nicotianae*, *F. graminearum*, *S. rolfsii*, *R. solani*, and *E. latusicollum*) were selected to assess the inhibitory effect of D7-8. The results showed that the growth of these six fungi was significantly inhibited by D7-8, with inhibition rates ranging from 42.4% to 56.8% D7-8 (Figure 1C–I) and the most substantial inhibition rate reaching 56.82 ± 3.90% against *P. nicotianae* (Figure 1C,E). These findings suggest that D7-8 can produce a broad spectrum of antifungal compounds, enabling it to inhibit the mycelial growth of various pathogenic fungi and highlighting its potential for further research.

Pot experiments were conducted with root irrigation to further assess the biocontrol potential of D7-8 (Figure 1J–L). The results demonstrated that most potatoes in the S group exhibited severe scab (Figure 1J), with a disease severity of 57.5% (Figure 1L). In contrast, the S+D group showed some scab presence (Figure 1L) but with a disease severity of only 25%, reflecting a relative efficacy of 56.52% (Figure 1M). These findings suggest that D7-8 effectively reduces the severity of potato common scab and holds promise as a biocontrol agent for managing this disease.

### 3.2. Phylogenomic Analysis of D7-8

To determine D7-8’s phylogeny and assess its taxonomic position within the bacterial kingdom, a preliminary phylogenetic tree was constructed based on *16S rRNA* and *rpoB* gene sequences. The evolutionary tree indicates that D7-8 shares a close relationship with *B. velezensis CBMB205* and is clustered in a single branch with *B. siamensis* KCTC (Figure S1). This analysis suggests that D7-8 is likely classified as *B. velezensis*.

### 3.3. Complete Genome Sequencing and Annotation of D7-8

**Figure 2:**
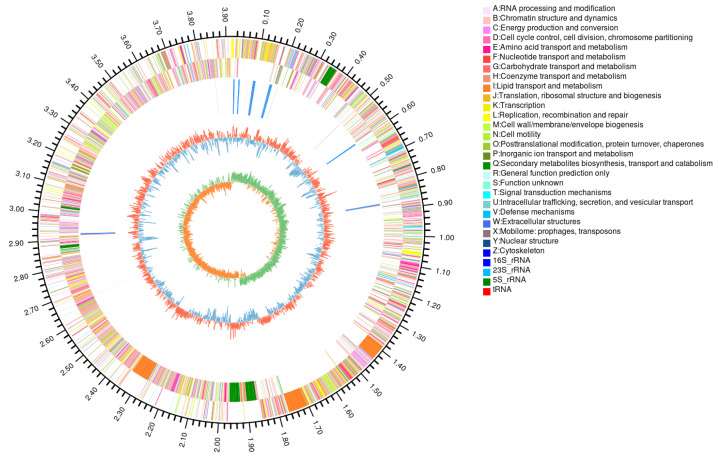
The genome of *Bacillus* D7-8. The circles from outermost to innermost indicate genome length, gene loci on the sense strand, gene loci on the antisense strand, ncRNA loci, GC content, and GC skew. The colors of genes on the sense and antisense strands represent their classification according to the COG database.

To gain better insights into the genetic characterization of D7-8 and its disease-preventive mechanisms, a complete genome was sequenced and assembled using PacBio and Illumina platforms. A total of 1.26 Gb and 1.31 Gb of raw sequencing data were obtained from the PacBio and Illumina platforms, respectively. The *contig* N50 of the PacBio reads was 9155 bp. After filtering out low-quality subreads, 45,363 high-quality long reads (0.42 Gb) and 7,874,262 short reads (1.19 Gb) were retained and subsequently assembled and polished to generate a complete genome for D7-8. The final assembly resulted in a single, closed circular chromosome without any gaps (Figure 2) and no plasmids were detected in the sequencing data. The BUSCO analysis of the genomic sequence suggested that the assembly was 100% complete (Table S2). The completed genome measured 3,916,873 bp with a GC content of 46.46%. Notably, the genome size of D7-8 was smaller than that of *Bacillus subtilis* 168 and *B. subtilis* TU-B-10, while its GC skew was slightly greater than that of these two reference genomes (Table S3). Repeat sequence analysis revealed that 0.27% of the D7-8 genome consists of repetitive elements, including 0.19% tandem repeats (TRs) and 0.08% interspersed repeats (IRs). Specifically, 38 TRs occupied a total of 7570 bp, while 47 IRs were classified into different repeat elements: 7 DNA transposons, 24 long interspersed nuclear elements (LINEs), 3 long terminal repeats (LTRs), and 13 short interspersed nuclear elements (SINEs), collectively spanning 3211 bp (Table S4).

In the complete genome of D7-8, 3926 genes were predicted, consisting of 3727 protein-coding genes with an average length of 929.16 bp (Table S5) and a total length of 3,462,987 bp, as well as 86 tRNAs, 27 rRNAs, and 86 sRNAs (Table S6). BUSCO analysis of the protein sequences suggested that the annotation completeness was 100% (Table S7). BLAST and KAAS searches were performed against multiple public databases, including Swiss-Prot, NR, KEGG, COG, and GO, to characterize gene functions. Of the 3727 protein-coding genes, 99.92% (3724) had functional annotations in at least one of these databases. The distribution of database hits was as follows: NR (3724 genes, 99.92%), Swiss-Prot (3524 genes, 94.55%), Pfam (3350 genes, 89.88%), COG (3089 genes, 82.88%), GO (2004 genes, 53.77%), and KEGG (2840 genes, 76.2%) (Figure 2; Table S8).

**Figure 3:**
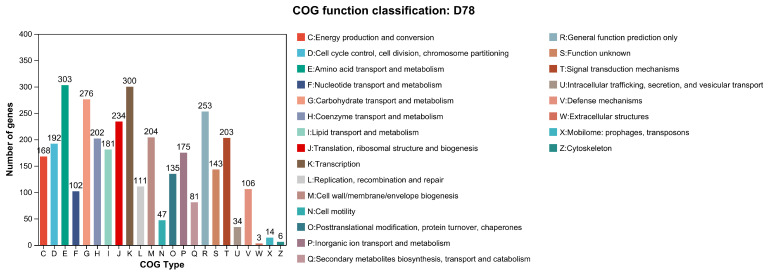
The COG function classification of D7-8 genes.

A total of 3089 genes were annotated into 23 COG categories, and the top three most represented COG functional groups were amino acid transport and metabolism (COG Type: E, 9.81%), transcription (COG Type: K, 9.71%), and carbohydrate transport and metabolism (COG Type: G, 8.93%) (Figure 3). GO enrichment revealed that 1023 genes were associated with cellular components, 1600 genes with molecular functions, and 1070 genes with biological processes (Table S9). Of the 2840 genes mapped to metabolic pathways in KEGG, the most abundant category was global and overview maps (844 genes), followed by carbohydrate metabolism (283 genes), amino acid metabolism (229 genes), and cofactor and vitamin metabolism (217 genes). Notably, dacC was identified as being involved in peptidoglycan biosynthesis (ko01100/ko00550), csn was linked to the chitosanase metabolic pathway (ko01100/ko00520), alsD and bdhA were identified as related to acetoin and 2,3-butanediol biosynthesis (ko01210/ko01110/ko00660/ko00650; ko00650/ko01100), and nasCDE was associated with nitrogen metabolism (ko01120/ko00910/ko01100) (Table S10).

### 3.4. Comparative Genomic Analysis of Bacillus Species

**Figure 4:**
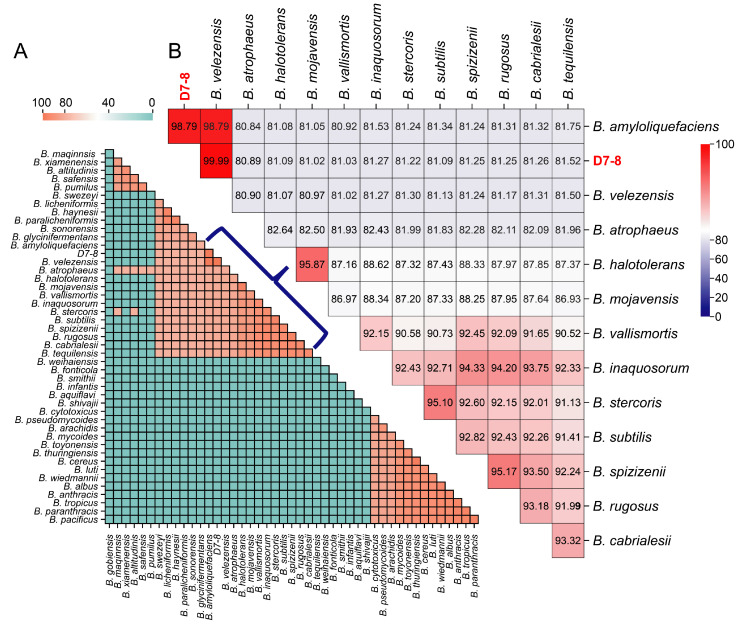
ANI analysis of 46 *Bacillus* genomes. (**A**) Heatmap of ANI values for 46 *Bacillus* genomes. The cyan squares represent the ANI values below 75. (**B**) Enlarged heatmap of ANI values of *Bacillus* species closely related to D7-8.

Based on accurate genomic data, a phylogenetic analysis of 31 housekeeping genes also showed that D7-8 was classified into the *Bacillus* genus and clustered in a distinct branch with *B. velezensis*, which exhibited a close relationship with *B. amyloliquefaciens* (Figure S2). To further confirm the taxonomic classification of D7-8, the Average Nucleotide Identity (ANI) was calculated among 46 high-quality reference genomes of *Bacillus* species, including D7-8 (Figure 4A). Cluster analysis of these genomes identified three distinct subgroups, with D7-8 belonging to the middle subset (Figure 4A). Within each subgroup, ANI values exceeded 80, highlighting the genetic diversity among *Bacillus* species at the nucleotide level. Compared to the model species *B. subtilis*, D7-8 exhibited a relatively low ANI score of 81.09, while *B. velezensis* and *B. subtilis* shared an ANI score of 81.13. In contrast, D7-8 displayed a high ANI similarity with *B. amyloliquefaciens* (98.79) and *B. velezensis* (99.99) (Figure 4B). These findings suggest that D7-8 is most closely related to *B. velezensis* or *B. amyloliquefaciens*.

**Figure 5:**
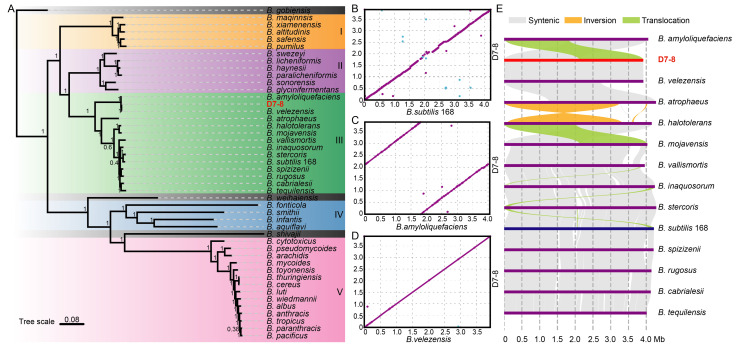
Phylogenetic analysis of 46 *Bacillus* species and synteny of the D7-8 genome with other *Bacillus* species: (**A**) phylogenetic analysis of 46 *Bacillus* species; (**B**) synteny between D7-8 and *B. subtilis* 168 using MUMMER; (**C**) synteny between D7-8 and *B. amyloliquefaciens* GKT04 using MUMMER; (**D**) synteny between D7-8 and *B. velezensis* FZB42 using MUMMER; and (**E**) structural variations characterization among 14 *Bacillus* species in subgroup III.

The large-scale phylogenetic analysis may provide more accurate details for classifying D7-8. The phylogenetic trees were constructed based on 493 single-copy genes from 46 *Bacillus* species using OrthoFinder v3.0.1b1. This phylogenetic tree clearly demonstrated that these 46 *Bacillus* species can be classified into five subgroups, with D7-8 clustered in subgroup III (Figure 5A). D7-8, *B. velezensis*, and *B. amyloliquefaciens* were further classified into a more refined subclass, with *B. amyloliquefaciens* serving as an outgroup to D7-8 and *B. velezensis*, indicating a closer evolutionary relationship between D7-8 and *B. velezensis* (Figure 5A). The syntenic analysis of interspecies genome sequences revealed that there were more non-syntenic regions between D7-8 and *B. subtilis* 168 than between D7-8 and *B. amyloliquefaciens* or *B. velezensis* (Figure 5B–D). D7-8 exhibited high synteny with *B. amyloliquefaciens*, but this synteny was classified into two regions (Figure 5C). In contrast, D7-8 was almost entirely syntenic with *B. velezensis* (Figure 5D). These results were also supported by the structural variation analysis using SyRI (Figure 5E). There was a large translocation of 1,792,115 bp or 1,788,528 bp between strain *B. amyloliquefaciens* and D7-8 (Figure 5E), which could explain the presence of two syntenic regions between these two *Bacillus* species (Figure 5C). A similar translocation was observed between *B. halotolerans* and *B. mojavensis*. This may be caused by genome assembly or the reticulate structure of bacterial chromosomes. Moreover, there were unaligned regions of 79,559 bp and 4874 bp in *B. amyloliquefaciens* and D7-8, respectively. There were 32,552 SNPs, 356 insertions, 401 deletions, one copy gain, three copy losses, and 83 highly diverged regions in the D7-8 genome compared with the *B. amyloliquefaciens* genome (Table S11). But alignment with *B. velezensis* showed only 411 bp of translocations, 76 SNPs, 5 insertions, 6 deletions, 1 copy loss, and 2 highly diverged regions in the D7-8 genome (Table S12), resulting in 3,916,057 bp (99.99% of the total genome) D7-8 being syntenic with the *B. velezensis* genome. For other alignments of interspecies, most regions were syntenic, but some inversions and translocations were also present, especially between *B. atrophaeus* and *B. halotolerans*, which exhibited a large inversion and a short inversion (Figure 5E). These structural variations also support the genetic diversity within the *Bacillus* genus, which may have caused their functional diversity. Most importantly, these results strongly support the classification of D7-8 as a species of *B. velezensis*.

### 3.5. Carbohydrate Metabolism and Biosynthesis Gene Clusters

The anabolic and catabolic capacities of carbohydrate metabolism suggested that the D7-8 genome contained 124 carbohydrate-active enzymes, which were categorized into 41 glycoside hydrolases (GHs), 40 glycosyl transferases (GTs), 32 carbohydrate esterases (CEs), 9 auxiliary activities (AAs), 3 polysaccharide lyases (PLs), and 2 carbohydrate-binding modules (CBMs). Furthermore, the GT class exhibited the highest representation from the GT2, GT4, and GT41 families, while the GH class was primarily composed of the GH1, GH13, and GH43 families. The CE1, CE4, and CE14 families dominated the CE class. The AA class was characterized by the AA7 and AA13 families. In contrast, the PL class contained only two families, PL1 and PL9, and the CBM class included just two families, CBM22 and CBM50 (Table S13).

**Figure 6:**
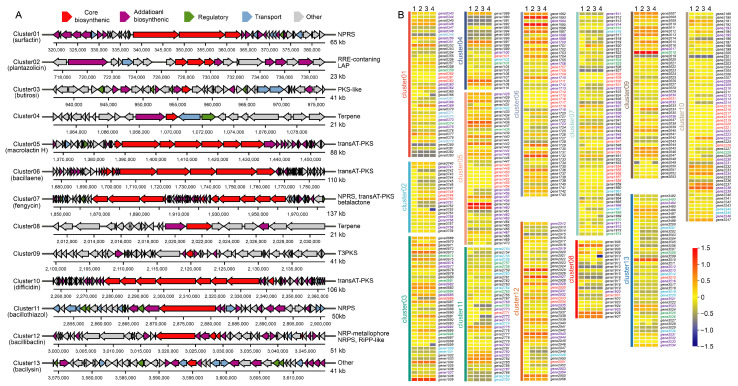
Biosynthetic gene clusters prediction of *B. velezensis* D7-8 genome and the expression pattern of genes involved in gene clusters: (**A**) the biosynthetic gene clusters in *B. velezensis* D-78 genome and (**B**) predicted expression profiles of a gene involved in the gene clusters from the genomes of *B. subtilis* 168, *B. amyloliquefaciens* GKT04, *B. velezensis* FZB42, and *B. velezensis* D7-8. The expression profiles were directly predicted using TXpredict software. 1: *B. subtilis* 168; 2: *B. amyloliquefaciens* GKT04; 3: *B. velezensis* FZB42; and 4: *B. velezensis* D7-8.

To further elucidate the fungal suppression mechanism of D7-8, secondary metabolite gene clusters were predicted using antiSMASH v7.0, revealing 13 gene clusters, including 9 known and 4 unknown clusters (Figure 6A). Among the identified clusters, four non-ribosomal peptide synthetase (NRPS)-type clusters were detected, including surfactin (91% similarity), fengycin (100% similarity), bacillibactin (100% similarity), and one unknown NRPS-type cluster. Three transAT-PKS-type clusters (macrolactin H, bacillaene, and difficidin) exhibited 100% similarity to known reference sequences. Furthermore, an RRE-containing cluster predicted to encode plantazolicin (91% similarity) and an other-type cluster predicted to encode bacilysin (100% similarity) were also identified (Figure 6A). The homologous genes (*gene0360*, *gene0361*, *gene0362*, *gene0363*) with WP_012116771.1, ABS72765.1, WP_012116774.1, and WP_012116775.1 were predicted to be associated with surfactin biosynthesis (*SrfABCD*) in the NR database, and five homologous genes (*PerR*, *PhoP*, *CcpA*, and *CsfB*) were classified as regulatory proteins related to bacillomycin biosynthesis. These findings highlight the genetic potential of D7-8 in producing bioactive secondary metabolites that may contribute to its antifungal activity.

On the heels of secondary metabolite gene cluster prediction, the genes involved in these clusters and their collinear genes in *B. subtilis* 168, *B. amyloliquefaciens* GKT04, and *B. velezensis* FZB42 were predicted using TXpredict software, a novel framework for predicting bacteria or archaea transcriptomes using only annotated genome sequences. According to the transcriptome prediction models provided by TXpredict, core biosynthetic genes and most auxiliary biosynthetic genes were typically either silenced or maintained at low expression levels. However, genes from *B. amyloliquefaciens* GKT04, *B. velezensis* FZB42, and D7-8 exhibited similar transcriptomic patterns (Figure 6B), consistent with their placement within a single evolutionary branch in the phylogenetic tree. Interestingly, distinct expression profiles were observed between *B. subtilis* and the other three *Bacillus* species, particularly in several gene clusters. Notable differences included *gene0364* and *gene0369* in cluster 01, *gene0748* and *gene0750* in cluster 02, *gene0986* in cluster 03, *gene1723* in cluster 06, *gene1837* in cluster 07, *gene1921* and *gene1925* in cluster 08, *gene2017* in cluster 09, *gene2209*, *gene2212*, *gene2224*, and *gene2239* in cluster 10, *gene2782* and *gene2783* in cluster 11, *gene2931* and *gene2951* in cluster 12, and *gene3531* and *gene3534* in cluster 13 (Figure 6B). This was especially notable for *gene0748* and *gene0750* in cluster 02, which were predicted to be involved in the biosynthesis of plantazolicin, an ultra-narrow-spectrum antibiotic. Additionally, genes associated with regulatory functions, transport mechanisms, and other modifications exhibited similar expression trends across *B. amyloliquefaciens* GKT04, *B. velezensis* FZB42, and D7-8, further supporting their shared evolutionary and functional characteristics (Figure 6B). We also observed that the expression profiles within the same *Bacillus* species were highly consistent in the transcriptomic model generated by TXpredict. This trend was preliminarily supported by the overall expression data of *B. velezensis* FZB42 and *B. velezensis* D7-8 (Table S14). These findings suggest that this transcriptomic model could serve as a potential tool for accurate bacterial identification. However, further validation of this hypothesis is necessary to confirm this approach.

### 3.6. Identification of Crude Extracts of Lipopeptides

**Figure 7:**
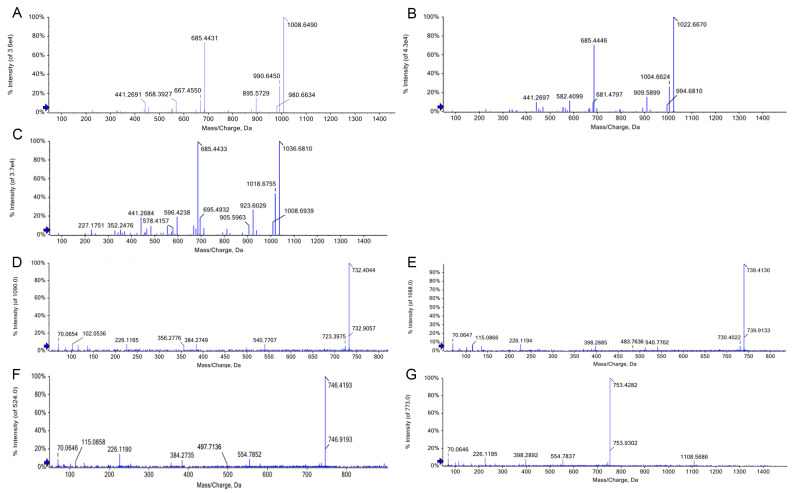
The MS/MS spectra of surfactin ions (**A**–**C**) and the MS/MS analysis of four fengycins (**D**–**F**). (**A**) *m*/*z* 1008.6490; (**B**) *m*/*z* 1022.6670; (**C**) *m*/*z* 1036.6810; (**D**) *m*/*z* 732.4044; (**E**) *m*/*z* 739.4130; (**F**) *m*/*z* 746.4193; and (**G**) *m*/*z* 753.4282.

Genome sequence analysis of *Bacillus velezensis* D7-8 revealed the presence of genes involved in the biosynthesis of various lipopeptides. The crude lipopeptide extract was analyzed to elucidate the composition of these lipopeptides. The UPLC-Q-Exactive HRMS analysis revealed two distinct signal peaks corresponding to lipopeptide families: surfactin (33–43 min) and fengycin (21–25 min). Further characterization of the surfactin family revealed [M+H]^+^ ions at *m*/*z* 1008.65 (surfactin A), 1022.67 (surfactin B), and 1036.67 (surfactin C), all sharing a standard core structure (β-OH fatty acid-Glu-Leu-Leu-Val-Asp-Leu-Leu) but differing in fatty acid chain lengths—13, 14, and 15 carbon atoms for surfactin A, B, and C, respectively. LC-MS analysis combined with reference data confirmed the structure of the ion at *m*/*z* 1036.67 (surfactin C), based on its characteristic fragment ions at *m*/*z* 923.60 (β-OH fatty acid-Glu-Leu-Leu-Asp-Val-Leu), 695.49 (β-OH fatty acid-Glu-Leu-Leu-Asp-Val), 685.44 (Val-Leu-Val-Asp-Leu-Leu), 596.42 (C15 β-OH fatty acid-Glu-Leu-Leu), 441.27 (Leu-Val-Asp-Leu), 352.24 (Asp-Leu-Leu), and 227.17 (Leu-Leu) (Figure 7A). Similarly, surfactin B (*m*/*z* 1022.67) displayed fragment ions at *m*/*z* 909.59 (β-OH fatty acid-Glu-Leu-Leu-Asp-Val-Leu), 695.49 (C14 β-OH fatty acid-Glu-Leu-Leu-Asp-Val), 685.44 (Val-Leu-Val-Asp-Leu-Leu), 681.47 (Glu-Leu-Leu-Val-Asp-Leu), 582.41 (Leu-Val-Asp-Leu-Leu), 441.27 (Leu-Val-Asp-Leu), and 227.17 (Leu-Leu) (Figure 7B). For surfactin A (*m*/*z* 1008.65), fragment ions were detected at *m*/*z* 895.57 (C13 β-OH fatty acid-Glu-Leu-Leu-Asp-Val-Leu), 685.44 (Val-Leu-Val-Asp-Leu-Leu), and 441.27 (Leu-Val-Asp-Leu) (Figure 7C). These data confirm the identification of surfactins A, B, and C in the crude extract.

Four fengycin derivatives, classified as fengycin A and fengycin B, were detected within the fengycin family. A characteristic fragment ion at *m*/*z* 540.77 was observed in the [M+2H]^2+^ ion peak at *m*/*z* 732.40, identifying this compound as C16 fengycin A (Figure 7D). Similarly, fragment ions at *m*/*z* 483.76 and 540.77 were detected in the [M+2H]^2+^ ion peak at *m*/*z* 739.41, corresponding to C17 fengycin A (Figure 7E). For the fengycin B variants, distinct fragment ions at *m*/*z* 497.71 and 554.78 were observed in the [M+2H]^2+^ ion peak at *m*/*z* 746.42, identifying the compound as C16 fengycin B (Figure 7F). Additionally, fragment ions at *m*/*z* 554.78 and 1108.57 were detected in the [M+2H]^2+^ ion peak at *m*/*z* 753.43, confirming the identification of C17 fengycin B (Figure 7G). These fengycin lipopeptides might contribute to antifungal activity against plant diseases such as potato common scab.

## 4. Discussion

Potato scab is a soil-borne disease caused by *Streptomyces* species [48]. The pathogen primarily affects the surface of the tuber, forming rough, scab-like lesions without penetrating deeply into the flesh. This disease significantly reduces tuber quality, making it a significant concern, particularly in the production of seed potatoes, which poses a serious challenge to the potato seed industry [49]. Timely irrigation, soil acidification, crop rotation, cultivation of resistant varieties, and the application of chemical agents are commonly used to manage potato scab [1,2]. However, these approaches have limitations, including the weak resistance of available cultivars, the short-term effectiveness of chemical treatments, environmental concerns, and the risk of developing pesticide resistance, all of which hinder the sustainable development of the potato industry. Biological control has emerged as a promising alternative for managing potato scab [50]. In this study, *B. velezensis* D7-8 showed good control ability, with a relative control efficiency of 56.52%. Compared to some microbial strains that have shown potential to inhibit disease in many studies, its efficacy is equivalent to or slightly lower than theirs. For example, Tao et al. reported that *B. velezensis* Y6 showed a higher efficacy of 88.10% in pot experiments, while Cao et al. reported that *B. atrophaeus* DX-9 showed an efficacy of 61.10% in pot experiments [51,52]. In the pot experiment conducted by Singhai et al., the control efficiency achieved by vermicompost treatment and *Pseudomonas* R1 reached 39.10% [18]. Additionally, a reported commercial microbial product consisting of *B*. *subtilis* strain znjdf1 and *Trichoderma harzianum* strain znlkhc1e exhibited a control efficiency of 50.15% in field trials [53]. Furthermore, in an experiment reported by Hassan et al., bioagents (*T. viride* and *Bacillus subtilis*) were isolated from the rhizosphere of potatoes, showing efficacies of 69.81% and 54.51%, respectively, against potato common scab under greenhouse conditions [54]. These findings show their effectiveness in reducing scab severity and provide a more environmentally friendly approach to disease management.

The *Bacillus* genus is a well-established bacterial agent recognized for its significant role in the biological control of plant diseases. These bacteria produce diverse secondary metabolites, including antibiotics, lipopeptides, enzymes, and other bioactive compounds, which contribute to their robust antagonistic properties against a broad spectrum of plant pathogens [55]. Antibiotics exhibit activity against fungi, bacteria, and viruses, enhancing the effectiveness of *Bacillus* species as biocontrol agents. The genome of *B. velezensis* D7-8 also contains antibiotic biosynthesis genes (Figure 3). In addition to direct antagonism, *Bacillus* species can induce systemic resistance in plants, thereby enhancing their ability to resist pathogens. This involves the activation of immune responses, such as the production of pathogenesis-related proteins and secondary metabolites, ultimately increasing plant resistance to subsequent pathogen attacks [56]. Furthermore, *Bacillus* species outcompete plant pathogens for essential nutrients and ecological niches in the rhizosphere, thereby reducing pathogen growth through rapid colonization and persistence in soil environments [57]. This competitive advantage helps maintain a balanced microbial ecosystem in the soil.

In addition to nutrient competition, *Bacillus* strains can degrade toxins produced by plant pathogens, neutralizing their detrimental effects on plants. Enzymatic degradation of these toxins not only prevents tissue damage but also promotes plant growth [58]. *Bacillus* species also produce volatile organic compounds such as acetoin and 2,3-butanediol, which possess antifungal and antibacterial properties. Genes responsible for the synthesis of these compounds have been identified in *B. velezensis* D7-8, suggesting that they may also be involved in the inhibition of pathogen growth and spore germination, further enhancing its biocontrol activity [59].

*Bacillus subtilis* is one of the most studied and commercially utilized species for biocontrol applications. It has been employed to manage various plant diseases, including Fusarium wilt, Rhizoctonia root rot, and Botrytis gray mold. The application of *B. subtilis* as a biopesticide has successfully reduced disease incidence and improved crop yields [60]. Other *Bacillus* species, such as *B. amyloliquefaciens*, *B. velezensis*, and *B. pumilus*, have also shown effective biocontrol properties. These species produce various lipopeptides and enzymes that inhibit the growth of pathogens, such as *Pythium*, *Phytophthora*, and *Sclerotinia* [61]. Additionally, *B. velezensis* K-9 has been shown to suppress potato common scab in field conditions [62]. They have proven effective in both soil and foliar applications, providing protection to both root and aerial parts of plants. Products from *Bacillus* species have been increasingly integrated into pathogen management strategies, reducing reliance on chemical pesticides. Their compatibility with other biological control agents and their reduced environmental impact make them a sustainable option for plant disease management [55].

But biocontrol also presents challenges, including inconsistent field performance, limited shelf life, and sensitivity to environmental conditions such as temperature and UV radiation. To overcome these limitations, enhancing the formulation of *Bacillus*-based products is crucial for improving their effectiveness and stability. Techniques such as microencapsulation and nanotechnology can protect *Bacillus* spores from adverse environmental conditions, thus extending their shelf life. Furthermore, developing advanced delivery systems, such as seed coatings and soil amendments, can enhance *Bacillus* colonization and persistence in the rhizosphere [58]. Advances in genetic engineering, especially CRISPR-based genome editing, offer further opportunities for genetic improvement to enhance the biocontrol potential of *Bacillus* species [63]. By introducing genes responsible for producing new antibiotics or improving the expression of existing metabolites, the biocontrol efficacy of *Bacillus* strains can be significantly improved [64]. Another approach is to select and optimize native strains that are adapted to local environmental conditions, which can further improve field performance [59]. But complete and accurate genetic information should be obtained before implementing genetic improvements to enhance the highly effective utilization of *Bacillus* species.

In the last two decades, more than 1400 *Bacillus* strains have been sequenced and released in the public database (https://www.ncbi.nlm.nih.gov, accessed on 10 February 2025), including complete reference genomes without any gaps. The genome of *B. velezensis* D7-8 used in this study is complete, high-quality, and gap-free (Figure 2). The wealth of genomic data provides valuable resources for research and the genetic improvement of *Bacillus*. However, the accurate identification of *Bacillus* species remains a challenge and a persistent issue in microbial taxonomy. The 16S ribosomal RNA gene is highly conserved across bacteria, with variable regions that can help distinguish species [65]. However, it is primarily useful for discriminating between bacterial genera rather than species under most conditions. The construction of phylogenetic trees by concatenating multiple genes, such as housekeeping genes or core genes, has been developed to identify microbes at the species level [66]. Corresponding software and scripts have also been developed, such as ACOPTools [67], PhyloSuite [68], and UBCG [69]. Average Nucleotide Identity (ANI) analysis is another method used to calculate the genomic similarity between whole genomes at the nucleotide level, and it is commonly employed for species identification. ANI offers high resolution in distinguishing between closely related species, with an ANI value of 95% considered the standard for defining species (Figure 4) [41]. This method of joint phylogenetic analysis provides accurate identification of microbes. The evolutionary tree is a powerful tool in biological research, allowing for the visualization of species relationships [31]. Closely related species are typically clustered within a single branch of a phylogenetic tree, reflecting their shared evolutionary history and the closer relationships among these species. *Bacillus* D7-8 was classified as *B. velezensis* using this method in this study (Figure 5), while structural variation analysis provided further insights into species relationships. This comparative approach enhances our understanding of microbial taxonomy and will aid in more precise bacterial identification in future studies.

From microbial identification to predictive modeling and image analysis, the methods of machine learning and deep learning are enhancing our understanding and management of microbial communities [70,71,72,73]. Still, only a small proportion of sequenced microbial species have been studied at the transcriptomic level, especially among uncultivated microbes derived from metagenomic data. With advances in sequencing technologies, more uncultivated microbial genomes have been assembled [74], but their gene expression patterns were not further studied due to the lack of RNA samples for gene cloning or RNA-seq analysis. Recently, various software programs and scripts have been developed to predict expression levels across different regions of these genes [45,75,76]. These tools can also be applied to studies of microbial genome cultures. These predictions require further model optimization to enhance accuracy, which will support further gene editing and the genetic improvement of microbial utilization.

The secondary metabolites produced by *Bacillus* species are key factors in inhibiting the growth of pathogenic bacteria, which play a significant role in biological control [77]. In this study, the lipopeptides produced by *Bacillus velezensis* D7-8 were identified, including surfactin A, B, and C. Based on characteristic fragment structure analysis, all three surfactin variants share a standard core structure (β-OH fatty acid-Glu-Leu-Leu-Val-Asp-Leu-Leu), differing only in their branched fatty acid chains (C13, C14, and C15, respectively). Fengycin derivatives were also identified, with C16–C17 fengycin A and C16–C17 fengycin B being the predominant products. Fengycin A and B were distinguished by their characteristic ion fragments, with [M+H]^+^ ions at *m*/*z* 1080/966 ([M+2H]^2+^ at *m*/*z* 540/483) for fengycin A and *m*/*z* 1108/984 ([M+2H]^2+^ at *m*/*z* 554/479) for fengycin B [78,79], which aligns with the experimental results of this study. Previous research has also demonstrated that surfactin plays a crucial role in inhibiting scab-causing pathogens [80], while fengycin is a potent antifungal agent against filamentous fungi. Fengycin’s mechanism of action involves its interaction with the phospholipid bilayer of fungal cell membranes, resulting in structural disruption and altered membrane permeability. This ultimately causes the leakage of cellular contents and cell death. Moreover, fengycin has been reported to induce systemic plant resistance by interacting with root cell membranes [81], despite genomic evidence suggesting the presence of bacillomycin D, which was not detected in this study. This discrepancy may be due to limitations in the elution procedure or the mass spectrometry parameters. Nevertheless, these findings highlight the rich and diverse lipopeptide production capacity of *B. velezensis* D7-8, contributing to its strong antibacterial activity and potential as a biological control agent.

## 5. Conclusions

In this study, a novel endophytic bacterium named *B. velezensis* D7-8 was isolated from the potato variety ZM1, which exhibited strong antagonistic activity against potato common scab and broad-spectrum fungistatic properties. Complete genomic and bioinformatics analyses of *B. velezensis* D7-8 indicated a close relationship between *B. velezensis* D7-8 and *B. velezensis* FZB42. Combined with HPLC-MS analysis, the results indicated that *B. velezensis* D7-8 has the ability to synthesize several lipopeptides, including surfactins and fengycins. The genome assembly of *B. velezensis* D7-8 presented here provides valuable genetic insights into its anti-filamentous fungal mechanisms, whichD7-8 may eventually help uncover the functional diversity of the *Bacillus* genus and its evolution in the bacterial kingdom. These findings lay a solid foundation for the development of *B. velezensis* D7-8 as a biocontrol agent against potato common scab in practical agricultural applications.

## Data Availability

The sequencing data for this project are available on the National Center for Biotechnology Information (NCBI) repository under BioProject PRJNA1196615, BioSample SAMN45598187, and Genomes CP179922.

## Supplementary Materials

The following supporting information can be downloaded at https://www.mdpi.com/article/10.3390/microorganisms13040770/s1; Figure S1: The phylogenetic analysis of D7-8 based on 16s sequences; Figure S2: The Phylogenetic analysis of D7-8 based on 31 housekeeping genes; Table S1: Inhibition rate of endophytic bacteria isolated from potato to Streptomyces acidiscabies; Table S2: The BUSCO valuation of D7-8 genome assembly; Table S3: Statistics comparison of information and characteristics of genomes of D7-8, spizizenii TU-B-10 and 168 strains; Table S4: Statistics of repeat sequences in D7-8 genome; Table S5: Statistics of protein-coding genes in D7-8 genome; Table S6: Statistics of small RNA in D7-8 genome; Table S7: The BUSCO valuation of D7-8 genome annotation; Table S8: Statistics of gene annotation in each public database; Table S9: The functional enrichment of genes from D7-8 genome in COG database; Table S10: The functional enrichment of genes from D7-8 genome in KEGG database; Table S11: Structural variation between the genome sequence of D7-8 and *B. amyloliquefaciens*; Table S12: Structural variation between the genome sequence of D7-8 and *B. velezensis*; Table S13: Statistics of gene related to carbohydrate-active enzymes in D7-8 genome; Table S14: The predicate expression pattern of genes in the biosynthenic gene clusters of D7-8 and their collinear genes in *B. subtilis* 168, *B. amyloliquefaciens*, and *B. velezensis*.
