# Supplementary figures and images for: Biocontrol Effect of Bacillus velezensis D7-8 on Potato Common Scab and Its Complete Genome Sequence Analysis
Source: Microorganisms. 2025 Mar 28;13(4):770. doi: 10.3390/microorganisms13040770 (PMC12029370; doi:10.3390/microorganisms13040770)

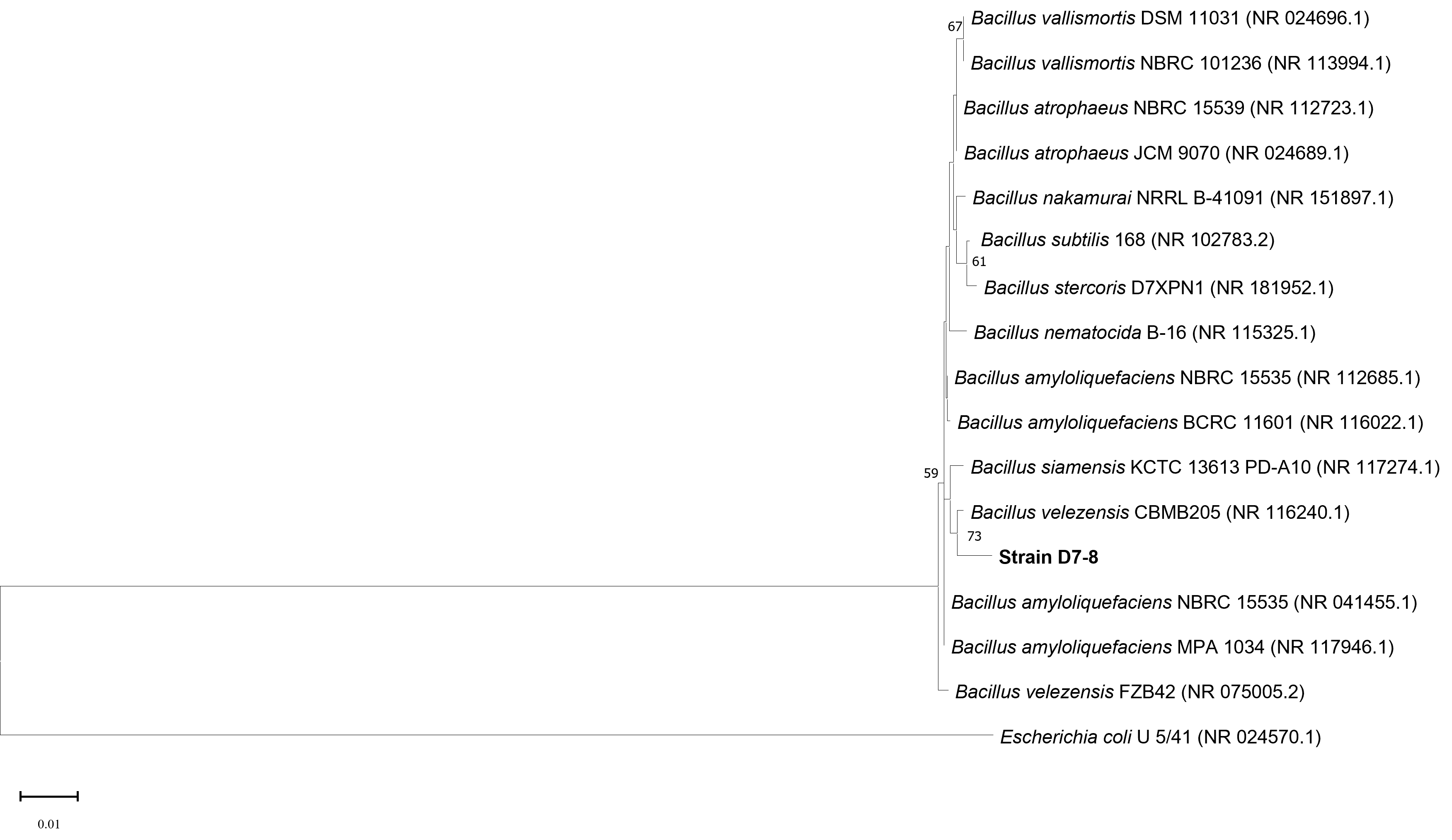

Supplement: Supplementary file 1 [file microorganisms-13-00770-s001.zip › Figure S1.png]
